# Acquired dysregulation of dopamine homeostasis reproduces features of Parkinson’s disease

**DOI:** 10.1038/s41531-020-00134-x

**Published:** 2020-11-13

**Authors:** Meghan L. Bucher, Caitlyn W. Barrett, Connor J. Moon, Amanda D. Mortimer, Edward A. Burton, J. Timothy Greenamyre, Teresa G. Hastings

**Affiliations:** 1 Pittsburgh Institute for Neurodegenerative Diseases, University of Pittsburgh, Pittsburgh, PA USA; 2 Department of Neurology, University of Pittsburgh School of Medicine, Pittsburgh, PA USA; 3 Department of Neuroscience, University of Pittsburgh School of Arts and Sciences, Pittsburgh, PA USA; 4 Geriatric Research, Education and Clinical Center, Pittsburgh VA Healthcare System, Pittsburgh, PA USA; 5 Present Address: Department of Environmental Health Sciences, Mailman School of Public Health, Columbia University, New York, NY USA

**Keywords:** Parkinson's disease, Transporters in the nervous system, Parkinson's disease

## Abstract

The catecholamine neurotransmitter dopamine has the potential to act as an endogenous neurotoxin when its vesicular sequestration is dysregulated. Despite postmortem analyses from patients with Parkinson’s disease that demonstrate decreased vesicular sequestration of dopamine with a corresponding increase in dopamine metabolism, dopamine’s contribution to nigrostriatal dopaminergic degeneration in Parkinson’s disease has been debated. Here, we present a new in vivo model demonstrating the induction of Parkinson’s disease-associated pathogenic mechanisms of degeneration resulting from acquired dysregulation of dopamine sequestration in nigrostriatal dopaminergic neurons in adult rats. Utilizing adeno-associated virus (serotype 2), viral-mediated small-hairpin RNA interference of endogenous vesicular monoamine transporter 2 (VMAT2) expression resulted in a loss of VMAT2 protein expression in transduced dopaminergic cell bodies in the substantia nigra with a corresponding loss of VMAT2 protein within the striatal terminals. The loss of VMAT2 resulted in an accumulation of cytosolic dopamine and subsequent increased dopamine metabolism, deficits in dopamine-mediated behaviors, and degeneration of nigrostriatal dopaminergic neurons that was rescued through reintroduction of exogenous VMAT2, demonstrating that the toxicity was specific to the loss of VMAT2. Analysis of parkinsonian pathogenic mechanisms of degeneration identified oxidative damage, activation of Parkinson’s disease-associated kinase LRRK2, and the formation of aberrant α-synuclein. This model demonstrates that a progressive acquired loss of VMAT2 expression in adulthood is sufficient to induce Parkinson’s disease-associated pathogenic mechanisms of degeneration and provides a new model to further investigate the consequences of cytosolic dopamine.

## Introduction

Parkinson’s disease is a progressive neurodegenerative disease characterized by motor deficits resulting from the degeneration of dopaminergic neurons in the nigrostriatal pathway. Postmortem analyses of brains from Parkinson’s patients show a loss of neuromelanin containing neuronal cell bodies in the substantia nigra and a progressive loss of dopaminergic terminals in the striatum. Within remaining neurons, there is evidence of neuronal dysfunction in the form of protein inclusions called Lewy bodies—a primary component of which is aggregated α-synuclein. Parkinson’s disease cases resulting solely from genetic factors are rare, and the majority of cases are believed to be the result of complex interactions between predisposition genes, a lifetime accumulation of environmental exposures, and deficits due to normal aging processes^1–3^. While multiple neuronal populations are affected in Parkinson’s disease, the nigrostriatal dopaminergic neurons appear to have increased vulnerability to degeneration and the loss of these neurons results in the cardinal motor symptoms that lead to a diagnosis of Parkinson’s disease. It is hypothesized that dopamine, the neurotransmitter these neurons use to regulate motor output, contributes to the vulnerability to degeneration; this is thought to be due to dopamine’s potential to act as an endogenous neurotoxin through two processes that generate reactive metabolites and reactive oxygen species: oxidation and enzymatic catabolism^4^.

Dopamine oxidation occurs either by autooxidation or enzymatic oxidation and generates the highly reactive and electrophilic dopamine quinone, producing either H_2_O_2_ or superoxide as a byproduct^5^. The dopamine quinone will bind to free and protein-bound nucleophilic cysteine, as well as highly nucleophilic selenocysteine found in selenoproteins, such as the mitochondrially-localized antioxidant selenoprotein glutathione peroxidase 4 (GPx4)^6^. The dopamine quinone has been shown to impair several neuronal processes with mitochondrial dysfunction being a common downstream consequence^6–10^. If dopamine does not undergo oxidation, it is susceptible to enzymatic catabolism, which generates the metabolite DOPAL while producing H_2_O_2_ as a byproduct^11^. Similarly to the dopamine quinone, upon accumulating in the cytosol, DOPAL will react with proteins and generate additional reactive oxygen species as a byproduct^12^. To minimize the accumulation of cytosolic dopamine to preserve neuronal health, there exist physical interactions between dopamine synthesis, packaging, and reuptake machinery^13,14^. Following synthesis in the cytosol or presynaptic terminal reuptake, dopamine is rapidly sequestered into synaptic vesicles by the vesicular monoamine transporter 2 (VMAT2) thereby isolating and stabilizing dopamine. However, there is significant evidence of VMAT2 deficiency in Parkinson’s disease, which implicates dysregulation of dopamine handling in the development and/or progression of the disease^15–17^.

Analysis from postmortem tissue from Parkinson’s disease patients revealed decreased expression of VMAT2, which suggests a deficiency in synaptic vesicle dopaminergic sequestration^15^. Furthermore, VMAT2 isolated from the striatal terminals from postmortem tissue of Parkinson’s patients shows an impaired rate of dopamine uptake, suggesting that in addition to a loss of VMAT2, the remaining VMAT2 is less efficient at sequestering dopamine^16^. Accordingly, analysis from postmortem tissue of Parkinson’s disease patients demonstrates increased cytosolic dopamine metabolism as evidenced by an increase in the amount of the dopamine metabolites DOPAC, HVA, and DOPAL relative to the amount of dopamine^16,18^. In addition, VMAT2 mRNA is decreased in circulating platelets from Parkinson’s disease patients, which suggests a systemic deficiency in VMAT2 involved in the pathogenesis of Parkinson’s disease^17^. Testing the hypothesis that a deficiency in VMAT2 contributes to the development of Parkinson’s disease, a genetic mouse model investigated the consequence of systemic VMAT2 deficiency and revealed the replication of motor and nonmotor Parkinson’s symptoms as well as age-dependent dopaminergic neurodegeneration^19,20^. Building on fundamental in vivo and in vitro experiments investigating the neurotoxic consequences of cytosolic dopamine, here we present a new in vivo model of acquired dopaminergic dysregulation through viral-mediated interference of VMAT2 expression in adult rats.

## Results

### RNA interference of VMAT2 expression in vitro

The vesicular monoamine transporter 2 (VMAT2) was targeted by ribonucleic acid interference using a short-hairpin RNA (shRNA) in a dopaminergic neuronal cell line (RCSN-3) derived from the midbrain of an adult rat as the initial step to test the feasibility of knocking-down VMAT2 expression in adult animals. Transfections with shVMAT2 were performed with coadministration of GFP to identify transfected cells, and efficiency of shVMAT2-mediated knockdown was quantified compared to cells transfected with a scramble shRNA (shControl). Analysis of VMAT2 protein immunoreactivity at 48 h post-transfection demonstrated a 41.4% decrease compared to shControl transfected cells (unpaired *t*-test, *n* = 37 cells in shControl treatment and 29 cells in shVMAT2 treatment, *p* < 0.0001) (Supplementary Fig. 1a, b). Analysis of VMAT2 mRNA by q-RT-PCR performed at the same timepoint identified a 46.0% decrease compared to shControl transfected cells (paired *t*-test, *n* = 4 experimental replicates, *p* = 0.0057) (Supplementary Fig. 1c). These data confirmed VMAT2 knockdown in vitro at both the mRNA and protein level by the shVMAT2 construct, suggesting the construct would be effective in vivo.

### Viral-mediated RNA interference of VMAT2 expression in vivo

**Fig. 1:**
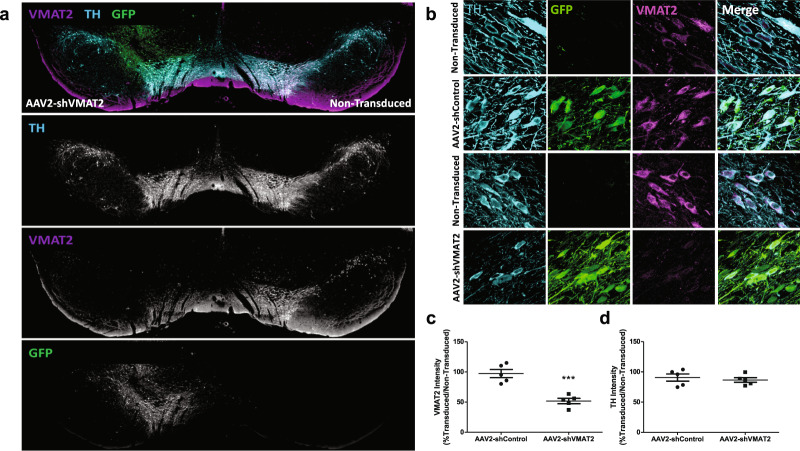
shRNA knockdown of endogenous VMAT2 in rat substantia nigra in vivo. **a** Representative image following unilateral AAV2-shVMAT2 injection to the substantia nigra (SN) demonstrating viral transduction, identified by GFP expression (green), and a loss of VMAT2 protein (purple) within TH-positive (blue) neurons. **b** 90x magnification confocal images of transduced TH-positive neurons in the SN following unilateral AAV2-shControl (top) and AAV2-shVMAT2 (bottom) with corresponding nontransduced contralateral hemispheres. **c** Quantification of VMAT2 immunoreactivity within AAV2-shControl and AAV2-shVMAT2 transduced TH-positive neurons expressed as a percentage of the immunoreactivity from internal control nontransduced TH-positive neurons in the contralateral hemisphere (unpaired *t*-test, *n* = 5 animals per treatment group, *p* = 0.0005). Each data point represents the average value for an individual animal calculated from at least 100 neurons per animal. **d** Quantification of TH immunoreactivity within AAV2-shControl and AAV2-shVMAT2 transduced TH-positive neurons expressed as a percentage of the immunoreactivity from internal control TH-positive neurons in the nontransduced contralateral hemisphere. (unpaired *t*-test, *n* = 5 animals, n.s.) Each data point represents the average value for an individual animal calculated from at least 100 neurons per animal. Error bars represent mean and standard error of the mean.

Following in vitro confirmation of VMAT2 knockdown, shVMAT2 was cloned into an AAV2 vector to express shVMAT2 under the U6 promoter, and green fluorescent protein (GFP) under the CMV promoter. AAV2-shVMAT2 virus was administered in vivo through unilateral injections in male adult Lewis rats targeting the dopaminergic neurons in the substantia nigra pars compacta (SNpc). Analysis performed at 6 weeks post-transduction demonstrated robust GFP immunoreactivity in tyrosine hydroxylase (TH) immunoreactive neurons in the SNpc indicating efficient viral transduction of dopaminergic (TH-positive) neurons (Fig. 1a, b). VMAT2 intensity within AAV2-shVMAT2 transduced TH-positive neurons (expressed as a percentage of internal control nontransduced neurons) was compared to AAV2-shControl transduced TH-positive neurons. AAV2-shVMAT2 transduced neurons demonstrated a 46.8% decrease in VMAT2 immunoreactivity compared to AAV2-shControl transduced neurons (unpaired *t*-test, *n* = 5 animals per treatment group, *p* = 0.0005) (Fig. 1c). However, TH immunoreactivity was maintained in AAV2-shVMAT2 transduced neurons, demonstrating that the decrease in protein immunoreactivity was specific to VMAT2 (unpaired *t*-test, *n* = 5 animals per treatment group, n.s.) (Fig. 1d). In addition, there was an equivalent loss of VMAT2 immunoreactivity following 6 weeks of viral transduction in female animals (Supplementary Fig. 2a) with maintained TH immunoreactivity (Supplementary Fig. 2b).

### Striatal dopamine dysregulation

**Fig. 2:**
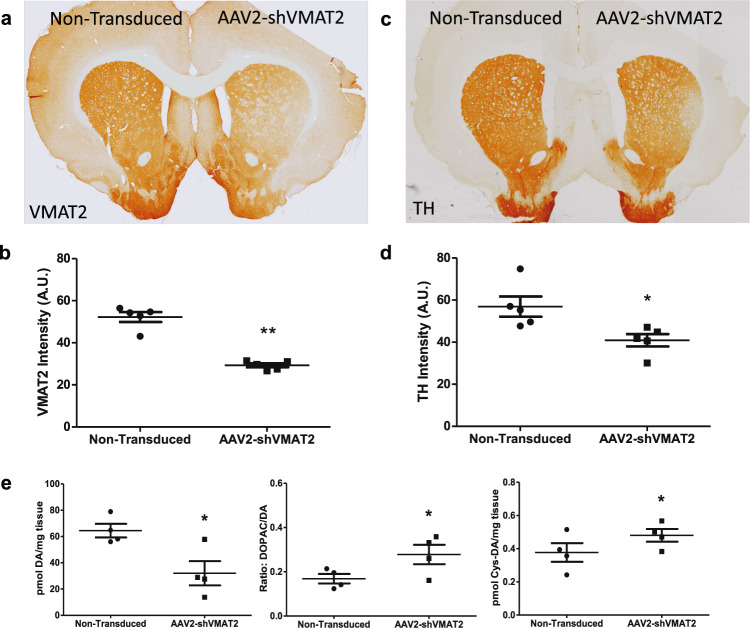
shRNA knockdown of endogenous VMAT2 results in dysregulated striatal dopamine. **a** VMAT2 expression in nigrostriatal dopaminergic terminals visualized by immunohistochemistry in the striatum following unilateral AAV2-shVMAT2 injection into the SN (transduced hemisphere identified by notch in cortex). **b** TH expression in nigrostriatal dopaminergic terminals visualized by immunohistochemistry in the striatum following unilateral AAV2-shVMAT2 injection into the SN (transduced hemisphere identified by notch in cortex). **c** Quantification of VMAT2 immunoreactivity in the dorsal striatum of the transduced hemisphere compared to the internal control nontransduced contralateral hemisphere (paired *t*-test, *n* = 5 animals, *p* = 0.0012). Each data point represents the average value for an individual animal calculated from three striatal sections per animal. **d** Quantification of TH immunoreactivity in the dorsal striatum of the transduced hemisphere compared to the internal control nontransduced contralateral hemisphere (paired *t*-test, *n* = 5 animals, *p* = 0.0215). Each data point represents the average value for an individual animal calculated from three striatal sections per animal. **e** Levels of dopamine (left), dopamine turnover as measured by a ratio of DOPAC:dopamine (middle), and dopamine oxidation as measured by protein cysteinyl-dopamine (right) at 8 weeks post-viral transduction. Measurements from the transduced striatal hemisphere were compared internally to the nontransduced contralateral hemisphere (paired *t*-test, *n* = 4 animals, *p* = 0.0433 for dopamine, *p* = 0.0209 for dopamine turnover, *p* = 0.0444 for cysteine-modified dopamine). Each data point represents the value per animal. Error bars represent mean and standard error of the mean.

In addition to a loss of VMAT2 protein in the TH-positive neuronal cell bodies of the SNpc, AAV2-shVMAT2 resulted in a corresponding loss of VMAT2 protein in the dorsal striatum 6 weeks post-transduction, as visualized by immunohistology with a colorgenic substrate (Fig. 2a). Striatal VMAT2 immunoreactivity demonstrated a 44.0% loss compared to the contralateral nontransduced hemisphere (paired *t*-test, *n* = 5 animals, *p* = 0.0012) (Fig. 2b). There was a focal loss of TH expression in the dorsal lateral striatum on the AAV2-shVMAT2 transduced hemisphere that is absent in the nontransduced hemisphere (Fig. 2c). This corresponded to a 28.1% loss of TH immunoreactivity in the AAV2-shVMAT2 group compared to the nontransduced hemisphere (paired *t*-test, *n* = 5 animals, *p* = 0.0215) (Fig. 2d).

We hypothesized that the decrease in striatal VMAT2 immunoreactivity would correspond with dysregulation of dopamine neurochemistry. To investigate this hypothesis, neurochemical analyses were performed at 8 weeks post-transduction in striatal tissue to measure dopamine and its metabolites. At 8 weeks post-transduction, dopamine was decreased in the transduced hemisphere by 50.4% compared to the nontransduced hemisphere (paired *t*-test, *n* = 4 animals, *p* = 0.0433) (Fig. 2e left); however, dopamine turnover indicated by the ratio of DOPAC to dopamine showed a significant 64.7% increase compared to the nontransduced hemisphere (paired *t*-test, *n* = 4 animals, *p* = 0.0338) (Fig. 2e middle). Protein cysteinyl-dopamine adducts, measured as an index of dopamine oxidation, were increased significantly by 27.4% in the transduced hemisphere compared to the nontransduced hemisphere (paired *t*-test, *n* = 4 animals, *p* = 0.0209) (Fig. 2e right).

### Dopaminergic neurotoxicity

**Fig. 3:**
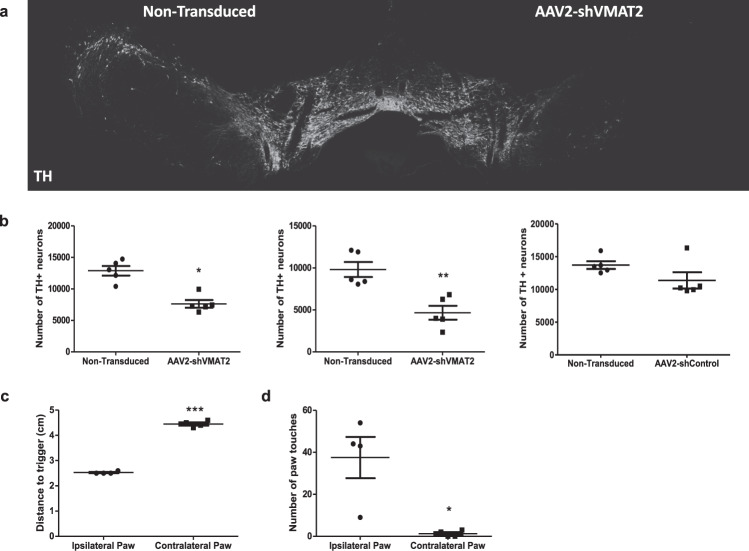
shRNA knockdown of endogenous VMAT2 results in degeneration in the substantia nigra. **a** Representative montage image of the SN immunolabeled for TH following unilateral AAV2-shVMAT2 transduction. **b** The number of TH-positive neuronal counts from the transduced hemisphere were compared to the internal control nontransduced contralateral hemisphere. Each data point represents the total number of TH-positive neurons in the SNpc of each hemisphere of an individual animal calculated from serial sections throughout the SN. Counts in males receiving unilateral AAV2-shVMAT2 (left) (paired *t*-test, *n* = 5 animals, *p* = 0.0177), females that received unilateral AAV2-shVMAT2 (middle) (paired *t*-test, *n* = 5 animals, *p* = 0.0046), and males receiving unilateral AAV2-shControl (right) (paired *t*-test, *n* = 5 animals, n.s.). **c** Postural instability test was performed 20 weeks post-transduction in animals with AAV2-shVMAT2 and contralateral AAV2-shControl injections. Contralateral paw refers to the paw contralateral to and therefore affected by the AAV2-shVMAT2 injection (paired *t*-test, *n* = 4 animals, *p* < 0.0001). Each data point represents the average distance to trigger for each forepaw in an individual calculated over three trials. **d** Cylinder test was performed at 20 weeks post-transduction in animals with AAV2-shVMAT2 and contralateral AAV2-shControl injections. The number of paw touches were counted during rearing behavior from three 5-min trials (paired *t*-test, *n* = 4 animals, *p* = 0.0301). Each data point represents the number of paw touches for each paw in an individual animal summed over 3 5-min trials. Error bars represent mean and standard error of the mean.

Given the evidence of increased cytosolic dopamine identified in Fig. 2, and previous research demonstrating that cytosolic dopamine can be neurotoxic, we next sought to identify whether VMAT2 knockdown resulted in dopaminergic neurotoxicity. A semi-automated fluorescent method of dopaminergic stereology that counts TH-positive neurons by identifying overlap between tyrosine hydroxylase (TH), a neuronal marker microtubule-associated protein 2 (MAP2), and the nuclear marker DAPI demonstrated that following 6 weeks of viral transduction, there was a loss of 40.8% of dopaminergic neurons in the transduced SNpc compared to the contralateral nontransduced hemisphere (paired *t*-test, *n* = 5 animals, *p* = 0.0177) (Fig. 3a, b left). Stereology performed in female animals under the same experimental conditions demonstrated a similar amount of neurodegeneration with 52.5% fewer dopaminergic neurons in the AAV2-shVMAT2 transduced hemisphere compared to the contralateral nontransduced hemisphere (paired *t*-test, *n* = 5 animals, *p* = 0.0046) (Fig. 3b middle). Stereological counts of TH-positive neurons from SNpc transduced with AAV2-shControl showed no significant difference in the number of TH-positive neurons in the transduced hemisphere compared to the nontransduced hemisphere, which is consistent with our previous report Zharikov et al. 2016^21^. Importantly, in a separate cohort of animals, stereological analysis performed at an earlier timepoint (4 weeks post-transduction) demonstrated no loss in TH-positive neurons, however a significant loss of TH-positive neurons was observed at 8 and 12 weeks post-transduction (Supplementary Fig. 3A). Even though there was no loss of TH-positive neurons in the SNpc at 4 weeks, there was a significant decrease in striatal dopamine levels (−41.72%) indicating that the knockdown of VMAT2 was altering terminal dopamine levels at earlier times points prior to degeneration (4 weeks paired *t*-test, *n* = 4 animals, *p* = 0.0085). Striatal dopamine levels showed a further decrease of −50.37% and −78.52% at 8 and 12 weeks, respectively (8 weeks paired *t*-test, *n* = 4 animals, *p* = 0.0433; 12 weeks paired *t*-test, *n* = 3 animals, *p* = 0.0391) (Supplementary Fig. 3B.)

To determine if the degeneration of nigrostriatal TH-positive neurons and dysregulation of striatal dopamine transmission resulted in motor deficits, two dopamine-mediated behavioral tests were performed in animals that received AAV2-shVMAT2 in one hemisphere and AAV2-shControl in the contralateral hemisphere. Behavioral tests showing robust differences between the right and left paws were performed at 20 weeks post-transduction. The postural instability test measures an animal’s ability to take a corrective step when placed off balance and has previously been shown to measure a Parkinsonian motor deficit rescuable by dopamine receptor agonist^22^. The forepaw contralateral to AAV2-shVMAT2 (affected paw) had a 78.0% increase in distance to trigger compared to the forepaw associated with the AAV2-shControl injected hemisphere, which performed at 2.5 cm (paired *t*-test, *n* = 4 animals, *p* < 0.0001) (Fig. 3c). As an additional measure, forepaw use was measured during the cylinder test, which has similarly shown decreases in rearing behavior in Parkinsonian conditions able to be rescued with dopamine receptor agonist^22^. Animals showed a reduction in paw preference for the affected forepaw contralateral to AAV2-shVMAT2 during rearing behavior. In contrast, animals showed a strong preference for the forepaw contralateral to AAV2-shControl indicating that its use was unaffected by the control viral injection (paired *t*-test, *n* = 4 animals, *p* = 0.0301) (Fig. 3d). These deficits in dopamine-mediated behaviors replicate behavioral phenotypes that have been observed in models of Parkinson’s disease^22^.

### Oxidative damage

To examine mechanisms associated with neurodegeneration following VMAT2 knockdown, specific pathogenic mechanisms and indices of neuronal degeneration were investigated. Because cytosolic dopamine is susceptible to processes that generate reactive oxygen species and oxidative stress, tissue was evaluated for the presence of oxidative damage to proteins. Protein modification by 3-nitrotyrosine (3NT) and 4-hydroxynonenal (4HNE) are markers of oxidative damage and have classically been used as an index of oxidative stress within neurons^23,24^. Decreased VMAT2 expression resulted in a 26.7% increase in 4HNE within transduced TH-positive neurons as compared to nontransduced TH-positive neurons in the SNpc (paired *t*-test, *n* = 9 animals, *p* = 0.0424), whereas neurons transduced with AAV2-shControl showed no increase in the amount of 4HNE (paired *t*-test, *n* = 3 animals, n.s.) (Supplementary Fig. 4a). Similarly, there was a 27.6% increase in 3NT within transduced TH-positive neurons as compared to nontransduced TH-positive neurons in the SNpc (paired *t*-test, *n* = 9 animals, *p* = 0.027), whereas neurons transduced with AAV2-shControl showed no increase in the amount of 3NT (paired *t*-test, *n* = 5 animals, n.s.) (Supplementary Fig. 4b). The increase in both 4HNE and 3NT levels suggests increased oxidative stress and oxidative modification of proteins.

### Induction of LRRK2 activity

**Fig. 4:**
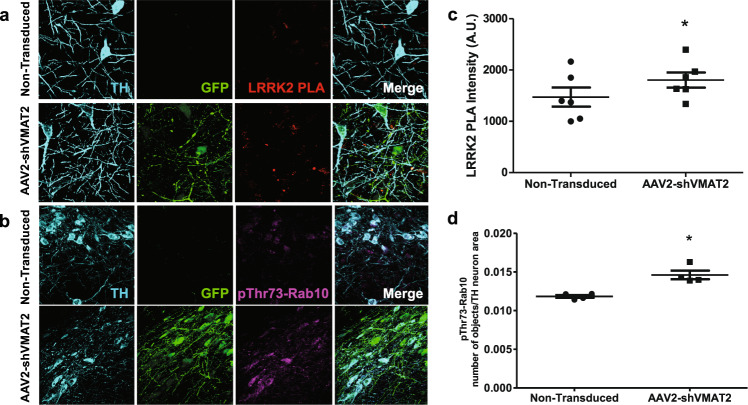
shRNA knockdown of endogenous VMAT2 results in increased LRRK2 activity. **a** 90x confocal images of TH-positive (blue) virally transduced GFP-expressing (green) neurons in the SN immunolabeled to identify activated LRRK2 by proximity ligation assay (PLA) (red). **b** Quantification of LRRK2 PLA immunoreactivity within transduced TH-positive neurons was compared to internal control nontransduced TH-positive neurons in the contralateral hemisphere (paired *t*-test, *n* = 6 animals, *p* = 0.0489). Each data point represents the average immunoreactivity of PLA signal in a TH neuron collected from at least 75 neurons per animal. **c** 90x confocal images of TH-positive (blue) virally transduced GFP-expressing (green) neurons in the SN immunolabeled to identify phosphorylated Rab10 (pThr73-Rab10) (blue). **d** Quantification was performed by counting the number of pThr73-Rab10-positive objects and controlling for TH neuron area. Number of objects/TH neuron area from AAV2-shVMAT2 transduced neurons were compared to internal control nontransduced TH-positive neurons in the contralateral hemisphere (paired *t*-test, *n* = 4 animals, *p* = 0.0313). Each data point represents the average number of objects in a TH neuron collected from at least 50 neurons per animal. Error bars represent mean and standard error of the mean.

Oxidative stress can induce activation of the Parkinson’s-associated kinase LRRK2^25^. Recently we have shown that proximity ligation assay (PLA) can be used to quantify the amount of autophosphorylated LRRK2, which is an indirect measure of LRRK2 activity^25^. The immunoreactivity of autophosphorylated LRRK2 was increased within transduced TH-positive neurons by 28.4% compared to nontransduced neurons in the SNpc (paired *t*-test, *n* = 4 animals, *p* = 0.025) (Fig. 4a, b). As an additional measure of LRRK2 activity, the amount of phosphorylated Rab10 (pThr73-Rab10)—a LRRK2 substrate—can be quantified. VMAT2 knockdown resulted in a 23.5% increase in the amount of pThr73-Rab within transduced TH-positive neurons as compared to nontransduced neurons in the SNpc (paired *t*-test, *n* = 4 animals, *p* = 0.0313) (Fig. 4c, d). Consistent with observations in males, analysis in female animals demonstrated an increase in both LRRK2 PLA immunoreactivity (paired *t*-test, *n* = 4 animals, *p* = 0.0225) (Supplementary Fig. 5a) and pThr73-Rab10 (paired *t*-test, *n* = 6 animals, *p* = 0.0091) (Supplementary Fig. 5b), whereas AAV2-shControl transduced TH-positive neurons demonstrated no significant difference in the amount of pThr73-Rab10 (paired *t*-test, *n* = 4 animals, n.s.) compared to the nontransduced hemisphere (Supplementary Fig. 5c).

### Formation of aberrant α-synuclein

**Fig. 5:**
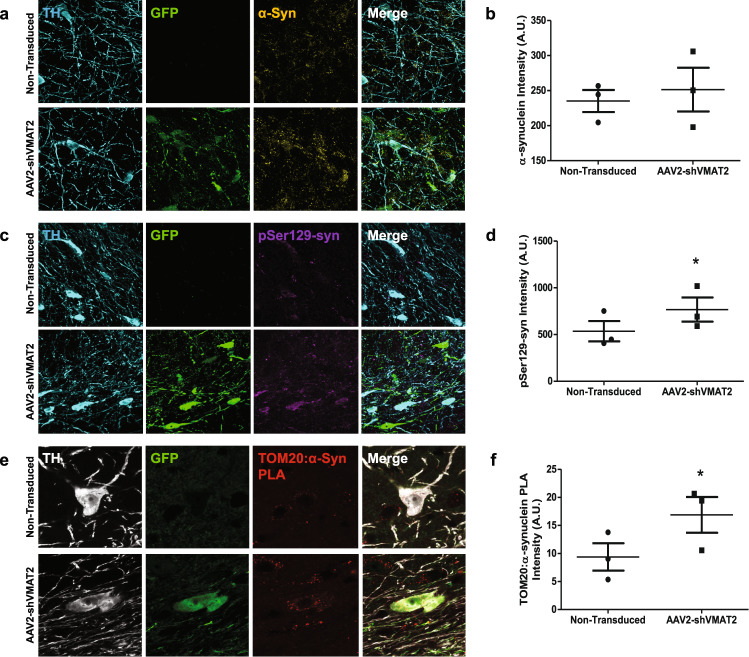
shRNA knockdown of endogenous VMAT2 results in aberrant α-synuclein. **a** 90x confocal images of TH-positive (blue) virally transduced GFP-expressing (green) neurons in the SN immunolabeled to identify α-synuclein (yellow). **b** Quantification of α-synuclein immunoreactivity within AAV2-shVMAT2 transduced TH-positive neurons were compared to internal control nontransduced TH-positive neurons in the contralateral hemisphere (paired *t*-test, *n* = 3 animals, n.s.). Each data point represents the average immunoreactivity for each animal calculated from at least 50 neurons per animal. **c** 90x confocal images of TH-positive (blue) virally transduced GFP-expressing (green) neurons in the SN immunolabeled to identify phosphorylated α-synuclein (pSer129-syn) (purple). **d** Quantification of pSer129-synuclein immunoreactivity within AAV2-shVMAT2 transduced TH-positive neurons was compared to internal control nontransduced TH-positive neurons from the contralateral hemisphere. (paired *t*-test, *n* = 3 animals, *p* = 0.014) Each data point represents the average immunoreactivity for each animal calculated from at least 75 neurons per animal. **e** 150x confocal images of TH-positive (white) virally transduced GFP-expressing (green) neurons in the SN immunolabeled to identify an interaction between α-synuclein and mitochondrial protein import protein TOM20 (red). **f** Quantification of fold change of TOM20:α-synuclein compared to nontransduced neurons (unpaired *t*-test, *n* = 3 animals, *p* = 0.026). Each data point represents the average fold change in PLA signal for each animal calculated from at least nine neurons per animal. Error bars represent mean and standard error of the mean.

One of the pathological hallmarks of Parkinson’s disease is the formation of protein aggregates, called Lewy bodies, that contain aberrant forms of α-synuclein. Although measurements of total α-synuclein immunoreactivity were not increased within transduced neurons (paired *t*-test, *n* = 3 animals, n.s.) (Fig. 5a, b), phosphorylated α-synuclein (pSer129-syn) was increased by 42.9% within transduced TH-positive neurons as compared to nontransduced neurons in the SNpc (paired t-test, *n* = 3 animals, *p* = 0.014) (Fig. 5c, d). In contrast, TH-positive neurons in the SNpc transduced with AAV2-shControl showed no significant differences in total α-synuclein (paired *t*-test, *n* = 4 animals, n.s.) or pSer129-syn compared to nontransduced TH-positive neurons in the SNpc (paired *t*-test, *n* = 4 animals, n.s.) (Supplementary Fig. 6a, b). Furthermore, pathogenic forms of α-synuclein (oligomeric, phosphorylated, or dopamine-modified) have been shown to disrupt mitochondrial protein import through an interaction with TOM20—a protein localized to the mitochondrial membrane^9^. This interaction can be measured by proximity ligation assay as previously described^9^. An interaction between α-synuclein and TOM20 was increased by 90.9% within AAV2-shVMAT2 transduced TH-positive neurons compared to nontransduced neurons (paired *t*-test, *n* = 3 animals, *p* = 0.0249) (Fig. 5e, f). Although there were no significant increases in total α-synuclein, the increase in pSer129-syn and the increase in interaction between an aberrant form of α-synuclein and TOM20 suggests pathogenic forms of α-synuclein were present following VMAT2 knockdown.

### Rescue by exogenous VMAT2

To confirm that neurodegeneration induced by AAV2-shVMAT2 was caused by loss of VMAT2, rather than viral transduction, off-target effects of the shRNA, or another artifact of the experimental approach, a second virus was constructed to reintroduce VMAT2 expression. Human VMAT2 contains one nucleotide difference in the region targeted by the rat shVMAT2 construct. Four additional silent mutations were introduced into human VMAT2 cDNA to make the transcript resistant to the shVMAT2 (knockdown resistant—kdrVMAT2). The construct, first tested in vitro, expresses human VMAT2 with a myc-DDK tag. Co-transfection of RCSN-3 cells with shVMAT2 and wild-type VMAT2 (wtVMAT2) did not rescue VMAT2 immunoreactivity. However, co-transfection with shVMAT2 and kdrVMAT2 restored VMAT2 immunoreactivity (Supplementary Fig. 7a). Using species-specific primers for VMAT2, analyses of human and rat mRNA revealed that co-transfection with shVMAT2 and kdrVMAT2 resulted in increased human VMAT2 mRNA, but not rat VMAT2 mRNA (one-way ANOVA with Tukey’s post-hoc test, *n* = 4–6 experimental replicates, *p < 0.05, **p < 0.01, ***p < 0.001) (Supplementary Fig. 7b, c). Collectively, these data demonstrate that the knockdown resistant construct is able to express exogenous VMAT2 in cells following shRNA targeting of endogenous VMAT2. An adeno-associated viral vector (AAV2-kdrVMAT2) was constructed to express the knockdown resistant version of VMAT2 in vivo.

**Fig. 6:**
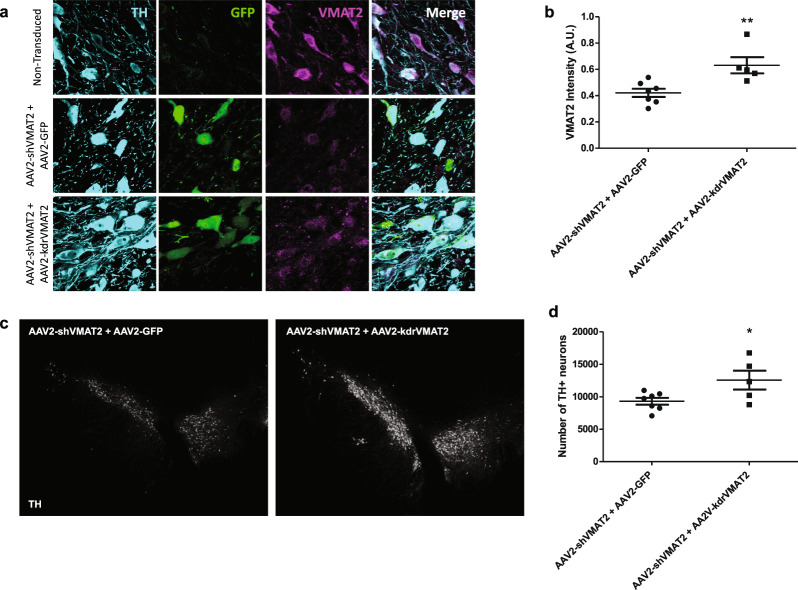
Neurodegeneration following endogenous VMAT2 knockdown is rescued by reintroduction of exogenous VMAT2. **a** Representative images following unilateral AAV2-shVMAT2 and AAV2-GFP co-injections (left panel) or AAV2-shVMAT2 + AAV2-kdrVMAT2 co-injections (right panel) to the SN demonstrating viral transduction identified by GFP expression (green), and VMAT2 protein (purple) within TH-positive (blue) neurons. **b** Representative 90x confocal images of nontransduced TH-positive neurons (top), AAV2-shVMAT2 and AAV2-GFP transduced TH-positive neurons (middle), and AAV2-shVMAT2 and AAV2-kdrVMAT2 transduced TH-positive neurons (bottom). **c** Quantification of VMAT2 immunoreactivity within AAV2-shVMAT2 and AAV2-GFP transduced TH-positive neurons compared to immunoreactivity within AAV2-shVMAT2 and AAV2-kdrVMAT2 transduced neurons (unpaired *t*-test, *n* = 5–7 animals per treatment group, *p* = 0.008). Each data point represents the average immunoreactivity for each animal calculated from at least 100 neurons per animal. **d** Representative SN images immunolabeled for TH following AAV2-shVMAT2 and AAV2-GFP co-injections (left) or AAV2-shVMAT2 and AAV2-kdrVMAT2 co-injections (right). **e** Quantification of stereological dopaminergic neuronal counts determined by colocalization of TH and Nissl (unpaired *t*-test, *n* = 5–7 animals per treatment group, *p* = 0.0146). Each data point represents the total number of TH-positive neurons in an individual animal calculated from serial sections. Error bars represent mean and standard error of the mean.

To test whether AAV2-kdrVMAT2 was able to reintroduce VMAT2 in vivo and protect against dopaminergic degeneration due to the loss of endogenous VMAT2, animals received co-injections of AAV2-shVMAT2 with either AAV2-kdrVMAT2 to reintroduce VMAT2 expression, or AAV2-GFP as a control that did not reintroduce VMAT2 (Fig. 6a). Immunohistochemistry was performed to detect TH, GFP, and VMAT2 immunoreactivity in each treatment group (Fig. 6b, c). Dopaminergic neurons expressing GFP in the AAV2-shVMAT2 + AAV2-kdrVMAT2 treatment group showed increased VMAT2 immunoreactivity compared to GFP-positive dopaminergic neurons in the AAV2-shVMAT2 + AAV2-GFP treatment group (unpaired *t*-test, *n* = 5–7 animals per treatment group, *p* = 0.008) (Fig. 6d). Fluorescent stereology was performed to count the number of dopaminergic neurons in the transduced and nontransduced hemispheres of each treatment group. The AAV2-shVMAT2 + AAV2-GFP treatment group had fewer TH-positive neurons in the transduced hemisphere in comparison to the number of TH-positive neurons in the transduced hemisphere of the AAV2-shVMAT2 + AAV2-GFP treatment group (unpaired *t*-test, *n* = 5–7 animals per treatment group, *p* = 0.0146) (Fig. 6e, f). These data suggest that reintroduction of exogenous VMAT2 by co-injecting AAV2-kdrVMAT2 prevented AAV2-shVMAT2-induced dopaminergic degeneration, indicating that the degeneration observed following a loss of endogenous VMAT2 (as seen in Fig. 2) was specific to the loss of VMAT2.

A summary of animal cohorts analyzed in this study is shown in Supplementary Table 2.

## Discussion

Although the contribution of dopamine to the pathogenesis of Parkinson’s disease has been controversial, there is a wealth of in vitro and in vivo literature demonstrating toxic consequences to dysregulated cytosolic dopamine and dopamine oxidation. Here, we demonstrate that acquired dopamine dysregulation in adulthood is sufficient to induce Parkinsonian degeneration in rats. Through viral-mediated RNA interference of VMAT2 expression, a decrease in VMAT2 immunoreactivity is observed in dopaminergic cell bodies in the substantia nigra pars compacta and within striatal terminals. As an index of cytosolic dopamine following VMAT2 knockdown, analysis of striatal tissue demonstrated a loss of dopamine with a corresponding increase in dopamine turnover, as measured by the ratio of DOPAC to dopamine, as well as an increase in dopamine quinone formation, as measured by protein cysteinyl-dopamine adducts.

**Fig. 7:**
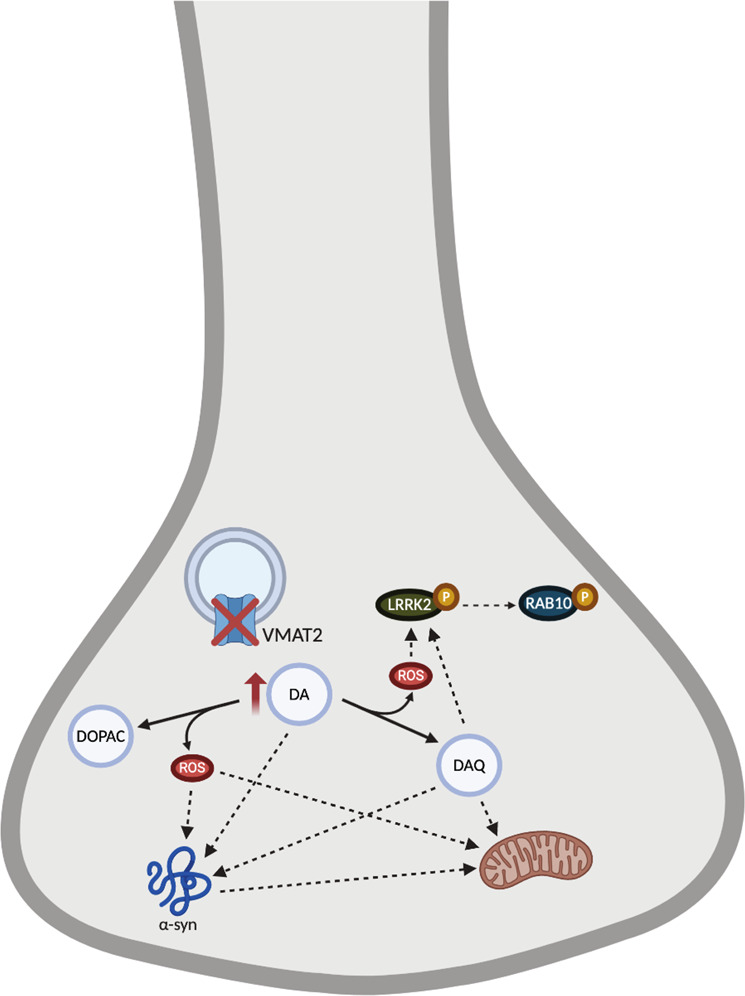
Summary of dopamine-mediated pathogenic mechanisms. A loss of VMAT2 results in increased cytosolic dopamine (DA) that undergoes catabolism to the metabolites, DOPAC, and the dopamine quinone (DAQ) while generating reactive oxygen species (ROS). ROS induce LRRK2 activity and downstream phosphorylation of LRRK2 substrates, and ROS and DA metabolites converge on α-synuclein resulting in aberrant α-synuclein that can impact mitochondrial functioning. Previous work has demonstrated interactions between DA and DAQ and α-synuclein, as well as interactions between DAQ and mitochondrial proteins. There are a number of additional interactions of cytosolic DA not depicted in this figure. This figure was created using BioRender.

Stereological analysis performed by identifying overlap of TH, microtubule-associated protein 2 (MAP2), and DAPI demonstrated a loss of dopaminergic neurons with a corresponding focal loss of TH in the dorsolateral striatum that is characteristic of dopaminergic degeneration. There was an extensive loss of MAP2 immunoreactivity across the substantia nigra, which is indicative of dopaminergic degeneration rather than only a loss of TH-phenotype. Furthermore, this degeneration was shown to be time dependent as analysis performed at earlier timepoints demonstrated dysregulation in dopamine packaging before the onset of degeneration. Replicating what has been previously demonstrated in rodent models of Parkinson’s disease, the nigrostriatal dopaminergic degeneration resulted in deficits in two dopamine-mediated behaviors commonly used to measure motor deficits. Furthermore, pathogenic mechanisms associated with Parkinsonian degeneration were evaluated within the remaining transduced TH-positive neurons and demonstrated an increase in LRRK2 activity as well as the formation of aberrant α-synuclein. Importantly, the neurodegeneration following knockdown of endogenous VMAT2 expression was able to be prevented by reintroducing exogenous VMAT2 expression. Our data suggest the induction of Parkinson’s-associated pathogenic mechanisms of degeneration as a result of direct interactions with cytosolic dopamine and its metabolites, and from the production of reactive oxygen species due to cytosolic dopamine as summarized in Fig. 7. Of note, this summary figure is a simplified representation, and given the evidence from previous publications cited within this paper, there are likely more interactions than depicted.

Previous research has investigated the consequences of genetically mediated dysregulation of dopamine handling. While VMAT2 knockout mice die shortly after birth, experiments performed in heterozygote VMAT2 knockout mice show increased susceptibility to dopaminergic toxins^26,27^. Additionally, VMAT2 hypomorph mice that express 5% of the normal amount of VMAT2 showed Parkinsonian dopaminergic degeneration in aged mice (18 months), as well as the replication of other features Parkinson’s disease^19,20,28^. In an animal model dysregulating dopamine homeostasis by targeting the plasmalemmal dopamine transporter (DAT), overexpression of DAT resulted in increased in dopamine metabolism associated with a loss of TH-positive neurons in the SNpc, motor deficits, and increased susceptibility to dopaminergic toxins^29^. Furthermore, Chen et al. (2008) demonstrated that introducing the DAT into non-dopaminergic striatal neurons that lack VMAT2, and therefore the machinery required for proper sequestration, resulted in oxidative damage and neurodegeneration, providing further evidence that dopamine without proper sequestration can induce non-dopaminergic neurotoxicity^30^. Given the nature of the mechanism of knockdown employed in our model, VMAT2 is lost gradually over time as shRNA blocks the translation of new VMAT2 and old VMAT2 is degraded. Therefore, in comparison to these previously established models with lifelong alterations in dopamine handling, the model we characterize here demonstrates the consequences of an acquired loss of VMAT2 expression in adulthood.

Until recently, analysis of the gene (SLC18A2) for VMAT2 within Parkinson’s disease cohorts was unable to conclude that variants in SLC18A2 result in increased likelihood of Parkinson’s disease^31^. However, a study published by Xiong et al. in 2016 identified two polymorphisms in SLC18A2 within a Parkinson’s disease cohort^32^. Furthermore, rare mutations in SLC18A2 result in infantile Parkinsonism^33–35^. In contrast, a number of studies have identified variants outside the coding region of SLC18A2 that increase expression of VMAT2 resulting in a gain-of-function and decreased likelihood of developing Parkinson’s disease^36–38^. These studies suggest that increased sequestration of dopamine can be protective against the development of Parkinson’s disease. Accordingly, when tested in vivo, mouse and drosophila models overexpressing VMAT2 demonstrate protection against dopaminergic neurotoxins and Parkinson’s disease-associated environmental toxins, further supporting the hypothesis that proper dopaminergic handling is required to maintain neuronal health^39–42^.

It is possible that independently of genetically derived alterations in VMAT2 expression, other factors may result in altered VMAT2 expression, thereby contributing to the propagation of the disease process through further oxidative stress and interactions with Parkinson’s disease-associated mechanisms of degeneration. Centrally, analysis from postmortem tissue identified decreased striatal VMAT2 immunoreactivity as well as decreased VMAT2 activity from isolated synaptic vesicles, whereas peripheral analysis revealed decreased VMAT2 mRNA in circulating platelets from Parkinson’s disease patients suggesting a possible systemic deficiency in Parkinson’s disease pathogenesis^15–18^. Previous work has also shown that the sequestration of dopamine is impaired downstream of Parkinson’s disease-associated environmental factors, and many studies have identified dysregulation of dopamine sequestration and release downstream of other Parkinson’s-associated genetic factors including misfolded α-synuclein, VPS35 variants, and LRRK2 variants^43–49^.

In addition, the dopamine quinone has also been shown to interact with Parkinson’s disease-associated chaperone protein DJ-1, and dopamine and its metabolic products have also been shown to interact with α-synuclein, which can cause downstream deficits in chaperone mediated autophagy, decreased glucocerebrosidase activity, damage to synaptic vesicles, and mitochondrial dysfunction^9,10,50–57^. Previous experiments have suggested dopamine mediates α-synuclein-induced toxicity both in cell culture and animal modeling experiments, and other recent studies have implicated calcium as a susceptibility factor mediating dopaminergic neuron vulnerability to α-synuclein^57–59^. Furthermore, Sang et al. (2007) demonstrated the bidirectional influence of VMAT on parkin-mediated parkinsonian phenotypes in drosophila. This study showed exacerbated parkinsonian toxicity in drosophila deficient in VMAT and with Parkinson’s-associated parkin mutations, whereas overexpression of VMAT attenuated mutant parkin-associated toxicity. These data suggest an interaction between cytosolic dopamine and parkin-mediated pathogenic mechanisms in Parkinson’s disease^60^. The cumulation of these studies demonstrate that impairment in dopamine sequestration is a convergent mechanism downstream of a variety of processes implicated in Parkinson’s disease, and that dopamine can contribute to the propagation of these same pathogenic mechanisms.

Although an increase in α-synuclein following VMAT2 knockdown has been previously reported, we did not detect a significant increase in total α-synuclein^19^. However, we detected an increase in both the amount of phosphorylated α-synuclein and the association between α-synuclein and TOM20, which we have shown blocks mitochondrial import^9^. Both of these observations suggest the presence of aberrant forms of α-synuclein, possibly including dopamine-modified α-synuclein^9^. It is likely that although the total amount of monomeric α-synuclein did not change in our model, given the abundance of evidence demonstrating an interaction between dopamine and α-synuclein, VMAT2 knockdown resulted in aberrant α-synuclein that contributed to the neurodegenerative process. This is further supported by the evidence of increased phosphorylated α-synuclein as the phosphorylation of α-synuclein at Ser129 has been extensively characterized for its pathogenicity^61^.

Recently, we have demonstrated an increase in activated LRRK2 as measured by proximity ligation assay in idiopathic Parkinson’s disease^25^. This increase corresponds with an increase in the amount of phosphorylated Rab10—a substrate for LRRK2. Taken together, these data suggest that increased LRRK2 activity is a common pathogenic mechanism in Parkinson’s disease. In the current study, we observed an increase in proximity ligation assay signal for LRRK2 and as an additional measure of LRRK2 activity, an increase in phosphorylated Rab10 following VMAT2 knockdown. The induction of LRRK2 activity downstream of cytosolic dopamine has previously been unreported in vivo. We have previously shown that LRRK2 activity can be induced by reactive oxygen species, and we hypothesized that oxidative stress produced as a consequence of increased cytosolic dopamine may induce LRRK2 activity^25^. Accordingly, our data demonstrated that following VMAT2 knockdown, there are increased oxidative modifications, which are an indirect marker of reactive oxygen species, and increased LRRK2 activity. We are the first to demonstrate the induction of LRRK2 activity downstream of dysregulated dopamine homeostasis in vivo.

It is important to mention that work published by Isingirini et al. did not detect degeneration following the conditional loss of VMAT2 in adult mice^62^. However, these experiments employed bilateral knockdown of VMAT2 resulting in a motor phenotype characterized by a decrease in total movement as early as 8 weeks following knockout, as well as a significant decrease in food and water intake with weight loss at 16 weeks. Interestingly, the decrease in movement, feeding, and drinking corresponded with a decrease in survival. It is possible that the bilateral loss of VMAT2 caused animals to succumb due to dehydration or starvation before neurodegeneration was observed. We hypothesize that a unilateral loss in VMAT2 expression in these mice may protect the animals against a loss of total movement, allowing them to survive for analysis at later timepoints, which may reveal dopaminergic neurodegeneration.

The experiments presented here provide the foundation for several follow-up studies that will further elucidate the toxic consequences of dysregulated dopamine homeostasis. As demonstrated in Fig. 1, there are a few neurons transduced with AAV2-shVMAT2 identified by GFP expression in the region of the substantia nigra pars compacta that lack detectable TH protein expression. It is possible that these are non-dopaminergic neurons transduced by the AAV2 viral vector or dopaminergic neurons that demonstrate an altered phenotype^63,64^. GFP-positive and TH-negative neurons were excluded from analysis in order to ensure the amount of proteins of interest quantified was only collected from confirmed transduced dopaminergic neurons. To that end, analysis within the transduced dopaminergic neurons in the substantia nigra pars compacta demonstrated no difference in TH immunoreactivity. By excluding the population of neurons expressing GFP but lacking TH, we may be reporting a conservative value of protein immunoreactivity changes as some of the GFP-positive and TH-negative neurons may have been dopaminergic neurons undergoing degeneration. However, additional analysis on this population of neurons may reveal more insight into the mechanisms active during degeneration.

In addition, to better understand the induction of pathogenic mechanisms, a thorough time-course can be investigated. Here, we have presented data from a number of timepoints post-transduction including 4, 6, 8, 12, and 20 weeks. Our initial analysis has demonstrated that the degeneration of dopaminergic neurons following a loss of VMAT2 indicates a trend toward progressive degeneration over time; however, additional studies are necessary to more clearly elucidate the temporal induction of pathogenic mechanisms. Additionally, we did not observe a consistent change in behavior at 6 weeks post-transduction likely due to compensatory mechanisms protecting extracellular dopamine levels in striatum with the loss of dopamine terminals. It is known that neurological deficits associated with dopamine loss do not appear until striatal dopamine levels are decreased by 80–85% of control levels. Given the biochemical analyses of striatal dopamine levels in this model, it is likely that behavioral deficits would have been observed as early as 12 weeks. Thus, in addition to the development of pathogenic mechanisms, the development of motor symptoms can be deeply characterized in future studies.

While we were able to identify indices of Parkinson’s-associated mechanisms of degeneration such as aberrant α-synuclein and induction of LRRK2 activity, additional experiments outside the scope of this manuscript are necessary to determine whether these mechanisms are causal or correlative to the degenerative process. Future experiments will investigate the role of these mechanisms in neurodegeneration by employing interventions targeting the individual processes and looking for neuroprotection. For example, a LRRK2 inhibitor could be administered to determine whether LRRK2 activation mediates the degenerative process downstream of dysregulated dopamine sequestration. Additionally, decreasing α-synuclein expression utilizing a viral approach previously published by our group would be useful in determining whether decreasing the amount of α-synuclein is be protective against dopamine-mediated degeneration^21,65^.

As an additional direction to understand the vulnerability to degeneration of the dopaminergic neurons in the substantia nigra, AAV2-shVMAT2 can be transduced within the ostensibly less-vulnerable dopaminergic neurons in the ventral tegmental area to evaluate whether Parkinson’s-associated mechanisms of degeneration are similarly induced and whether neurodegeneration occurs. Previous work has shown resistance to dopaminergic degeneration within the ventral tegmental area of mice deficient in VMAT2, which mimics data from human studies demonstrating resistance to degeneration in the ventral tegmental area of Parkinson’s disease patients^28,66^. Investigating how the dopaminergic neurons in the ventral tegmental area respond differently to cytosolic dopamine may provide insight both into why these neurons are resistant to degeneration and which pathogenic mechanisms are necessary mediators of dopamine-induced toxicity.

As summarized above, our model of acquired dysregulation of dopamine homeostasis can be used to further understand the mechanisms by which dopamine to acts an endogenous neurotoxin and investigate how these processes relate to Parkinson’s disease pathogenesis. There are several benefits to the viral-mediated knockdown of VMAT2 employed in this study. The virus can be injected directly into the brain of adult animals targeting specific areas rather than having global knockdown from birth. A unilateral injection of the virus also has the advantage of an internal control on the contralateral hemisphere. The extent of knockdown can be modulated through diluting the virus to introduce fewer viral particles or adjusting timepoint for experiments, and with neurodegeneration observed as early as 6 weeks post-transduction, this allows for more feasible testing of therapeutic interventions on a faster timescale. Furthermore, the acquired loss of VMAT2 prevents the development of compensatory mechanisms characteristic of genetic models, and models an acute loss of VMAT2 activity in adulthood. Collectively, this model of dysregulated dopamine sequestration has demonstrated that cytosolic dopamine is sufficient to induce Parkinson’s disease-associated pathogenic mechanisms of neurodegeneration. The implications of which further suggests a therapeutic potential for VMAT2 in the treatment of Parkinson’s disease.

## Methods

### Cell culture

Cell culture experiments were performed in RCSN-3 cells: an immortalized dopaminergic neuronal cell line derived from the substantia nigra of an adult rat. RCSN-3 cells were obtained through an agreement with the University of South Florida and the University of Chile and maintained in media (DMEM, 10% BS, 2.5% FBS, 40 mg/l GS) in an incubator at 37 °C with 100% humidity and an atmosphere of 10% CO_2_. Predesigned small-hairpin ribonucleic acid (shRNA) constructs against rat vesicular monoamine transporter 2 (VMAT2) were obtained from Origene and the shRNA with best knockdown efficiency was chosen for viral production (Origene catalog number TG709635). A scramble control shRNA construct was provided from Invitrogen and used for in vitro experiments. Cells were transfected with a construct for green fluorescent protein (GFP) and shRNA constructs using Lipofectamine LTX and Plus Reagent and collected at 48 h post-transfection for RNA and protein analysis. Immunocytochemistry was used to visualize protein immunoreactivity and protein quantification was performed on images obtained on an Olympus IXB1 confocal in Fluoview software. Regions of interest (ROIs) were drawn around GFP-positive cells. RNA was isolated utilizing the RNeasy Mini Kit (Qiagen catalog number 74104). For q-RT-PCR analysis, cDNA was generated with the iScript cDNA Synthesis Kit (Bio-rad catalog number 1708890). Primers were obtained from Real Time Primers (Elkins Park, PA). A list of primers with sequences can be found in Supplementary Table 1. Site-directed mutagenesis was performed to construct the shRNA resistant VMAT2 (kdrVMAT2) construct utilizing NEBaseChanger Site-Directed Mutagenesis Kit (New England BioLabs catalog number E0554S).

### Viral vectors

The oligonucleotide encoding the small-hairpin ribonucleic acid for VMAT2 is targeted against the following nucleotide sequence: [TCA][ACA][GTT][ATG][TTT][GCC][TTC][TCC][AGC].

The nucleotide sequence in the knockdown resistant VMAT2 viral vector is: [TCT][ACG][ATC][ATG][TTC][GCC][TTC][TCC][AGC]. The oligonucleotide encoding the shRNA construct with optimal VMAT2 knockdown (Origene, catalog number TG709635) was annealed into the BamHI/EcoRI sites of pAAV-D(+)-U6-siRNA-CMV-GFP^21^. Adeno-associated virus (serotype 2) vector particles containing viral genomes derived from the pAAV vector were produced by the Penn Vector Core. The resulting shVMAT2 vector (AAV2-shVMAT2) expresses the shRNA construct under the human U6 promoter and eGFP under the CMV promoter with a titer of 3.56 × 10^13^ genome copies/mL. A previously established non-targeting shRNA control vector based on the same vector genome was used as a control^21^. The knockdown resistant VMAT2 construct expresses human VMAT2 with four silent mutations and a myc-DDK tag. This construct was cloned into pZac2.1 plasmid and the AAV2-kdrVMAT2 virus was produced by the Penn Vector Core with a titer of 2.00 × 10^12^ genome copies/mL. A control virus overexpressing GFP (AAV2-GFP) was ordered from Penn Vector Core with a titer of 2.00 × 10^12^ genome copies/mL. The AAV2-shVMAT2 virus was diluted to equal titer (2.00 × 10^12^ genome copies/mL) in sterile PBS for rescue experiments. For rescue experiments, fresh viral aliquots and dilutions as necessary were prepared on the day of the surgery. Viruses were diluted to the same viral titer in sterile PBS and equal proportions of each virus were mixed thoroughly. Animals received 2 μL of mixed viral preparations (AAV2-GFP + AAV2-shVMAT2 or AAV2-shVMAT2 + AAV2-kdrVMAT2) equivalent to 1 μL each of the individual viruses injected as described below. In the study presented here, the knockdown resistant virus was administered at the same time as the knockdown virus, which models the protective replacement of exogenous VMAT2 expression as endogenous VMAT2 is lost. This study design was chosen as an initial proof of principle experiment to demonstrate the specificity of neurodegeneration as a result of VMAT2 knockdown and to circumvent possible confounding variables or issues surrounding multiple viral injections into the brain.

### Animals and stereotaxic surgery

Adult (≥3 months) male and female Lewis rats were obtained from Envigo (Indianapolis, Indiana, USA). Rats were singly housed in temperature-controlled conditions under a 12:12 light-dark cycle with ad libitum access to food and water. All experiments were approved by the University of Pittsburgh Institutional Animal Care and Use Committee.

Stereotaxic surgery was performed to deliver virus to the substantia nigra (Bregma −5.8 AP, +/−2.2 ML, −8.4 DV). Animals were deeply anesthetized under isoflurane (2–4%) for the entirety of the surgery. Two microliter of virus was infused over a 10-min period (0.2 uL/min). Animals were closely monitored on a heated surface during recovery of surgery. Animals received analgesia in the form of ketoprofen (3.0 mg/kg s.c.) with the first dose presurgery and 2×/day for 3 days following surgery. Animals were sacrificed by transcardial perfusion with phosphate buffered saline followed by 4% paraformaldehyde. The brains were harvested and sectioning was performed on a freezing-stage microtome (ThermoFisher HM 450 sliding microtome) at 35 µm. Sections were maintained in cryoprotectant at −20 °C until analysis.

### Behavioral testing

Deficits in dopamine-mediated behaviors were evaluated by two previously established tests of dopamine-mediated deficits in Parkinson’s disease models—the postural instability test and the cylinder test^22^. For the postural instability test, the distance the animal is moved to trigger a corrective step was measured for each forepaw. The task was performed in triplicate and an average in performance was calculated per day. For the cylinder test, the animal was placed in a clear cylinder and allowed to freely rear for a 5-min period. The cylinder test was repeated three times across a 2-week period for each animal to aggregate total number of paw touches.

### Histology and proximity ligation assay

Immunohistochemistry was performed on free-floating sections before mounting on glass slides for imaging. Primary antibodies used for immunohistochemical analysis are listed in Table 1.

**Table 1:** Antibodies used for immunohistochemistry.

Protein	Host	Company and catalog #	Dilution
GFP	Mouse	Millipore MAB3850	1:2000
TH	Rabbit	Millipore AB152	1:2000
VMAT2	Goat	Santa Cruz sc-7721	1:1000
4HNE	Rabbit	Abcam AB46545	1:500
3NT	Mouse	Santa Cruz sc-32757	1:500
α-synuclein	Mouse	BD Biosciences 610787	1:2000
Phosphorylated α-synuclein	Rabbit	Abcam AB1253	1:2000
Phosphorylated Rab10	Rabbit	Abcam ab230261	1:1000
MAP2	Mouse	Millipore MAB378	1:2000
TOM20	Rabbit	Santa Cruz sc-11415	1:1000
LRRK2	Mouse	UC Davis N241A/34	1:500
Phosphorylated LRRK2	Rabbit	Abcam AB203181	1:500

Proximity ligation assay was analyzed utilizing PLA probes (Duolink; Sigma–Aldrich catalog numbers: DUO92001, DUO92001, DUO92004, DUO92005) and visualized with orange (Duolink; Sigma–Aldrich catalog number DUO92007-30RXN) and far-red (Duolink; Sigma–Aldrich DUO92013-30RXN) kits.

For quantification of immunofluorescence, confocal images of both immunohistochemistry and proximity ligation assay were taken on an Olympus IXB1 confocal. Intensity measurements were obtained in Fluoview software by circling ROIs around TH-positive neurons in the nontransduced hemisphere and TH- and GFP-positive neurons in the transduced hemisphere while blinded to the protein of interest. Intensity values of the protein of interest were compared between transduced and nontransduced TH-positive neurons. Number of objects analysis was performed in Nikon Elements software by circling ROIs around TH-positive neurons in the nontransduced hemisphere and TH- and GFP-positive neurons in the transduced hemisphere while blinded to the protein of interest. Within ROIs, a threshold was applied to the protein of interest in order to achieve unbiased counts of objects. Images to visualize DAB immunoreactivity were taken on an Olympus BX61VS microscope and analyzed for pixel saturation in CellSens software.

### Unbiased stereology

Stereology was initially performed on serial sections from the midbrain following a protocol adapted from Tapias et al. 2014^67^. Briefly, tissue was immunolabeled for TH, MAP2, and DAPI and imaged at ×20 on a Nikon90i fluorescent microscope in the Center for Biologic Imaging at the University of Pittsburgh. Images were analyzed in Nikon Elements software counting the number of dopaminergic neurons determined by overlap between DAPI, MAP2, and TH within a region of interest defining the substantia nigra pars compacta as previously described^67^. Subsequent experiments were performed with a modified method of stereology utilizing overlap between TH and fluorescent Nissl (NeuroTrace 647, Life Technologies) within the substantia nigra pars compacta as previously described^68,69^. These images were obtained on an Olympus BX61VS slide scanning microscope, and analysis was performed utilizing Nikon Elements software.

### Biochemical analysis

Animals were sacrificed at 8 weeks post-transduction via transcardial perfusion with phosphate buffered saline (PBS). Following PBS perfusion, the striatal tissue was dissected, weighed (12–30 mg), and rapidly frozen on dry ice. Tissues were stored at −80 °C until used for the analyses of free and protein-bound cysteinyl catechols and parent catechol levels. Tissue was analyzed as previously described^70^. Striatal tissue was homogenized in 0.1 M perchloric acid. Homogenates were centrifuged (34,000 × *g* for 20 min) to separate the acid precipitated protein from the acid-soluble components and stored at −80 until analysis. The acid-soluble component was filtered (Spin-X centrifuge tube filter, 0.22 µm; Costar) and analyzed by HPLC-ED (high performance liquid chromatography with electrochemical detection). The protein pellet was washed by resuspension and recentrifugation in 0.1 M perchloric acid containing 0.2 mM sodium bisulfite and then subjected to acid hydrolysis in 6 N HCl as we previously described^70^. Following drying and resuspension, the catechol moieties were extracted with alumina and the cysteinyl catechol were analyzed using HPLC-ED (Waters Alliance 2695 HPLC system with 2465 Electrochemical detector; Microsorb-MV column (C18; 4.6 × 250 mm; Agilent)). The mobile phase contained 0.05 M sodium phosphate, 0.01 M citric acid, 0.01 M sodium acetate, 0.32 mM sodium octyl sulfate, and 0.1 mM EDTA, 10% (vol/vol) methanol, and was titrated to pH 2.5 with HCl. Compounds were identified by their elution positions and quantified by comparison to catechol standards.

### Statistics and data reporting

Data are presented as individual data points corresponding to an individual animal and “n” equal to the number of animals unless otherwise specified. Graphs demonstrate mean and standard error of the mean. Paired *t*-tests were used to evaluate within animal protein immunoreactivity, neurochemical measurements, neuronal counts, and behavior. Unpaired *t*-tests were used to compare viral treatment groups. One-way ANOVA with Tukey’s post-hoc test was performed to evaluate mRNA.

### Study approval

Experimental approval was obtained through the Institutional Animal Care and Use Committee at the University of Pittsburgh.

### Reporting summary

Further information on research design is available in the Nature Research Reporting Summary linked to this article.

## Supplementary information

Supplementary Figures and Tables

Reporting Summary FLAT

## Data Availability

The data generated during and/or analyzed during the current study are available within the paper and supplementary files.
