# Supplementary material for: Acquired dysregulation of dopamine homeostasis reproduces features of Parkinson’s disease
Source: NPJ Parkinsons Dis. 2020 Nov 13;6:34. doi: 10.1038/s41531-020-00134-x (PMC7666186; doi:10.1038/s41531-020-00134-x)
Supplement: Supplementary file 1 — Supplementary Figures and Tables [file 41531_2020_134_MOESM1_ESM.pdf]

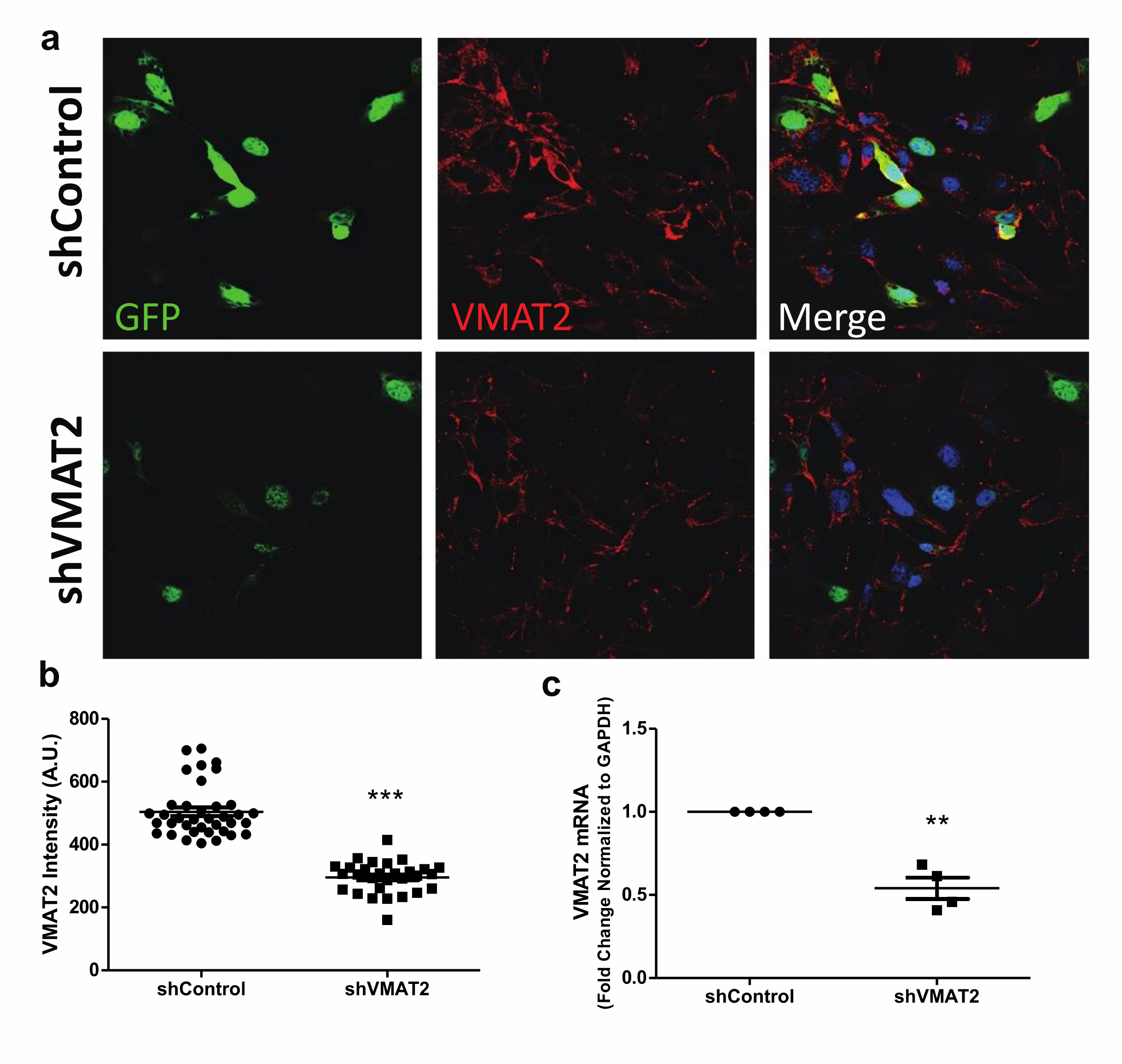


Supplementary Figure 1. shRNA-mediated VMAT2 knock-down *in vitro*. A. 60x confocal images of immunocytochemistry in RCSN-3 cells co-transfected with shControl and GFP (top) or shVMAT2 and GFP (bottom) immunolabeled for GFP (green), VMAT2 (red), and DAPI (blue). B. Quantification of VMAT2 immunoreactivity from GFP expressing cells demonstrated a 41.4% loss of VMAT2 protein in shVMAT2 transfected cells compared to shControl transfected cells (unpaired t-test, n=37 cells in shControl treatment and 29 cells in shVMAT2 treatment, p<0.0001). C. VMAT2 mRNA normalized to GAPDH analyzed by qtPCR was decreased by 46.0% in shVMAT2 transfected cells compared to shControl transfected cells (paired t-test, n=4 experimental replicates, p=0.0057).

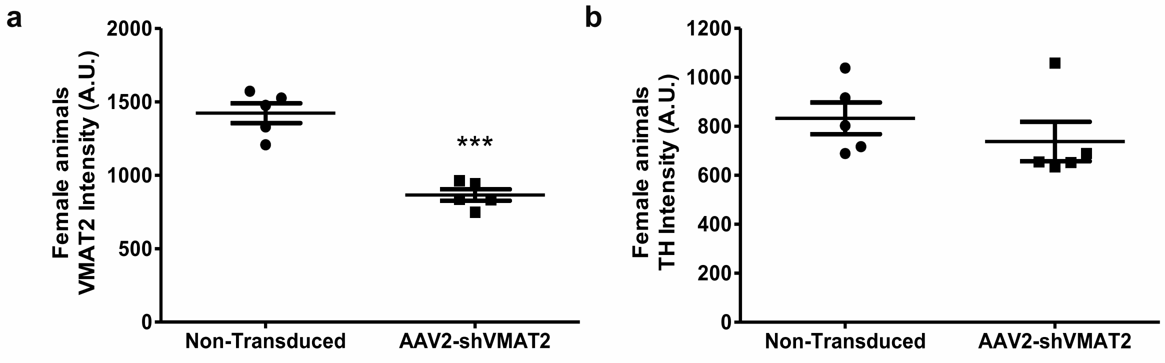


Supplementary Figure 2. shRNA knock-down of endogenous VMAT2 in female rat SN. A. Quantification of VMAT2 immunoreactivity within AAV2-shVMAT2 transduced TH-positive neurons and non-transduced TH-positive neurons in the contralateral hemisphere (paired t-test, n=5 animals per treatment group, $p<0.001$). Each data point represents the average value for an individual animal calculated from at least 100 neurons per animal. B. Quantification of TH immunoreactivity within AAV2-shVMAT2 transduced TH-positive neurons and non-transduced TH-positive neurons in the contralateral hemisphere. (paired t-test, n=5 animals, n.s.) Each data point represents the average value for an individual animal calculated from at least 100 neurons per animal.

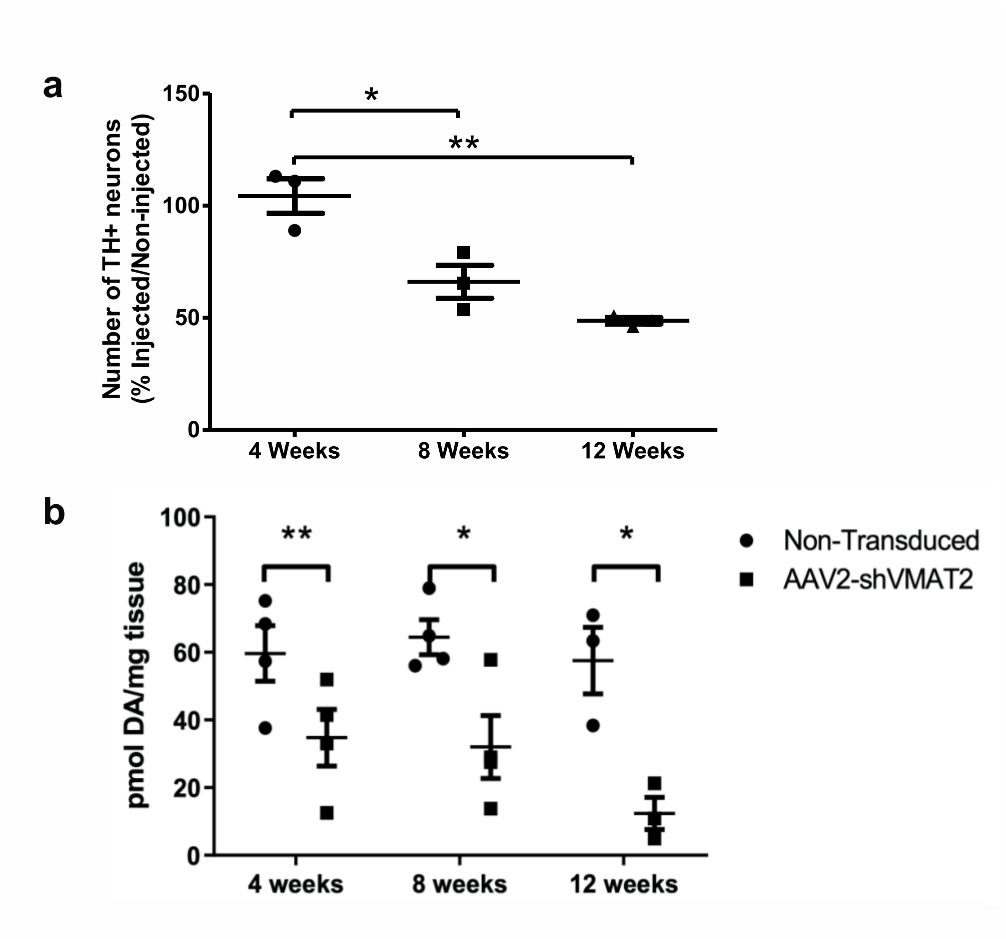


Supplementary Figure 3. shRNA knock-down of endogenous VMAT2 results in time-dependent degeneration and dysregulated striatal dopamine. A. Stereological counts of TH-positive neurons at three time-points following AAV2-shVMAT2 transduction expressed as the percent of TH-positive neurons in the transduced hemisphere neurons compared to the contralateral non-transduced hemisphere (One-way ANOVA with Tukey's post-hoc test, n=3 animals per time-point, *p<0.05, **p<0.01). B. Levels of dopamine at 4, 8, and 12 weeks post-transduction in non-transduced and AAV2-shVMAT2 transduced hemispheres. Measurements from the transduced striatal hemisphere were compared internally to the non-transduced contralateral hemisphere. Each data point represents the value per animal. (paired t-test, n=4 animals, 4 weeks p<0.01; 8 weeks p<0.05; 12 weeks p<0.05).

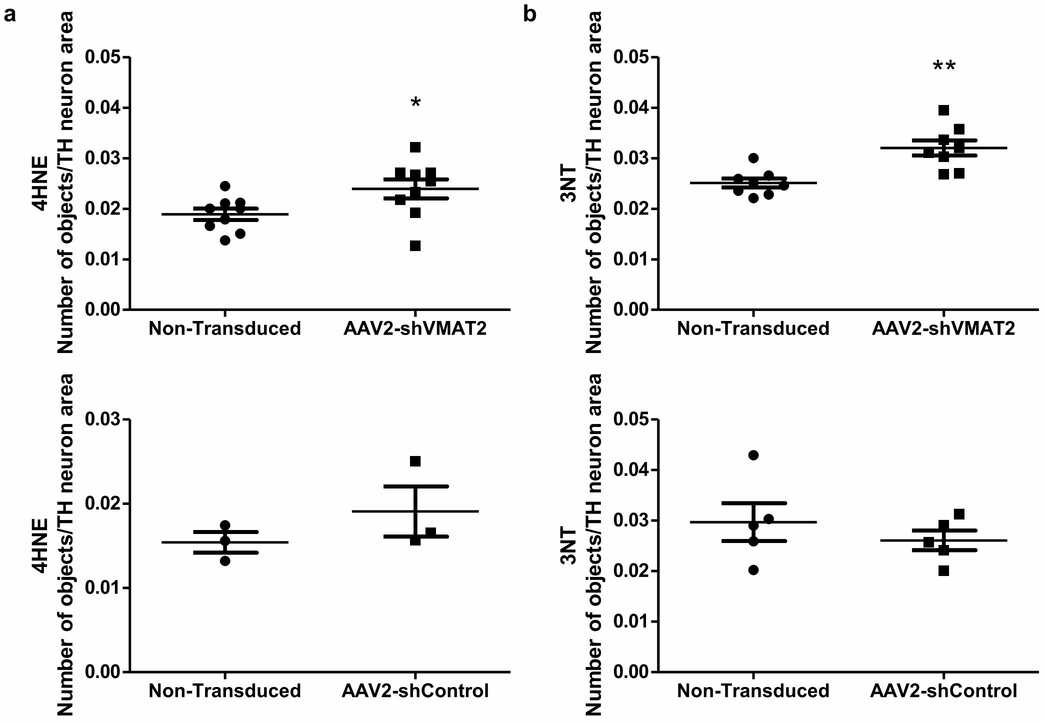


Supplementary Figure 4. shRNA knock-down of endogenous VMAT2 results in oxidative damage. A. Quantification of the amount of 4HNE within AAV2-shVMAT2 transduced TH-positive neurons (top) were compared to non-transduced TH-positive neurons from the internal control contralateral hemisphere (paired t-test, n=9 animals, p=0.0424). Quantification of the amount of 4HNE within AAV2-shControl transduced TH-positive neurons (bottom) were compared to non-transduced TH-positive neurons from the internal control contralateral hemisphere (paired t-test, n=3 animals, n.s.). Each data point represents the average number of objects in a TH neuron collected from at least 50 neurons per animal. B. Quantification of the amount of 3NT within AAV2-shVMAT2 transduced TH-positive neurons (top) were compared to non-transduced TH-positive neurons from the internal control contralateral hemisphere (paired t-test, n=9, p=0.0027). Quantification of the amount of 3NT within AAV2-shControl transduced TH-positive neurons (bottom) were compared to non-transduced TH-positive neurons from the internal control contralateral hemisphere (paired t-test, n=5 animals, n.s.). Each data point represents the average number of objects in a TH neuron collected from at least 50 neurons per animal.

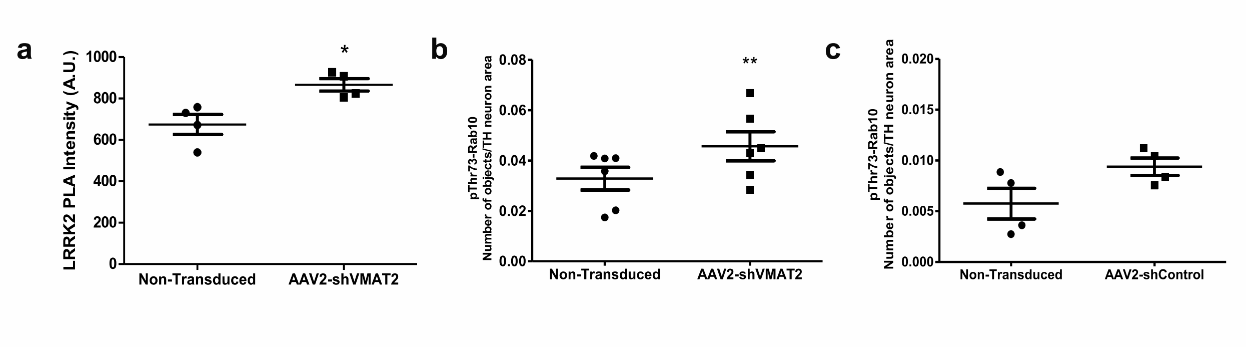


Supplementary Figure 5. shRNA knock-down of endogenous VMAT2 results in increased LRRK2 activity in female animals. A. Quantification of LRRK2 PLA immunoreactivity within transduced TH-positive neurons was compared to internal control non-transduced TH-positive neurons in the contralateral hemisphere in female animals (paired t-test, n=4 animals, p=0.0225). Each data point represents the average immunoreactivity of PLA signal in a TH neuron collected from at least 75 neurons per animal. B. Quantification of the amount of pThr73-Rab10 within AAV2-shVMAT2 transduced TH-positive neurons compared to non-transduced TH-positive neurons from the internal control contralateral hemisphere in female animals (paired t-test, n=6 animals, p=0.0091). Each data point represents the average number of objects in a TH neuron collected from at least 50 neurons per animal. C. Quantification of the amount of pThr73-Rab10 within AAV2-shControl transduced TH-positive neurons compared to non-transduced TH-positive neurons from the internal control contralateral hemisphere (paired t-test, n=4, n.s.). Each data point represents the average number of objects in a TH neuron collected from at least 50 neurons per animal.

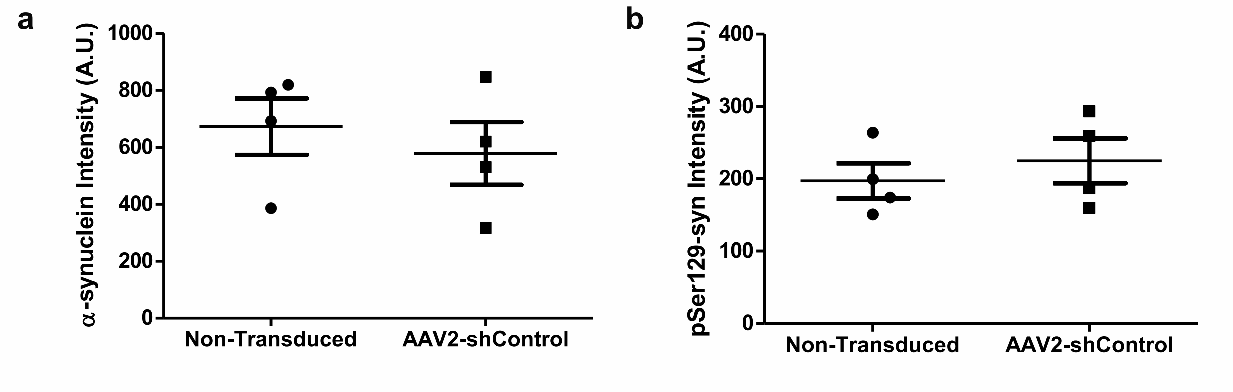


Supplementary Figure 6. Analysis of α-synuclein in AAV2-shControl transduced substantia nigra. A. Quantification of α-synuclein immunoreactivity within AAV2-shControl transduced TH-positive neurons were compared to internal control non-transduced TH-positive neurons in the contralateral hemisphere (paired t-test, n=4 animals, n.s.). Each data point represents the average immunoreactivity for each animal calculated from at least 50 neurons per animal. B. Quantification of phosphorylated α-synuclein (pSer129-syn) immunoreactivity within AAV2-shControl transduced TH-positive neurons was compared to internal control non-transduced TH-positive neurons from the contralateral hemisphere (paired t-test, n=4 animals, n.s.). Each data point represents the average immunoreactivity for each animal calculated from at least 50 neurons per animal.

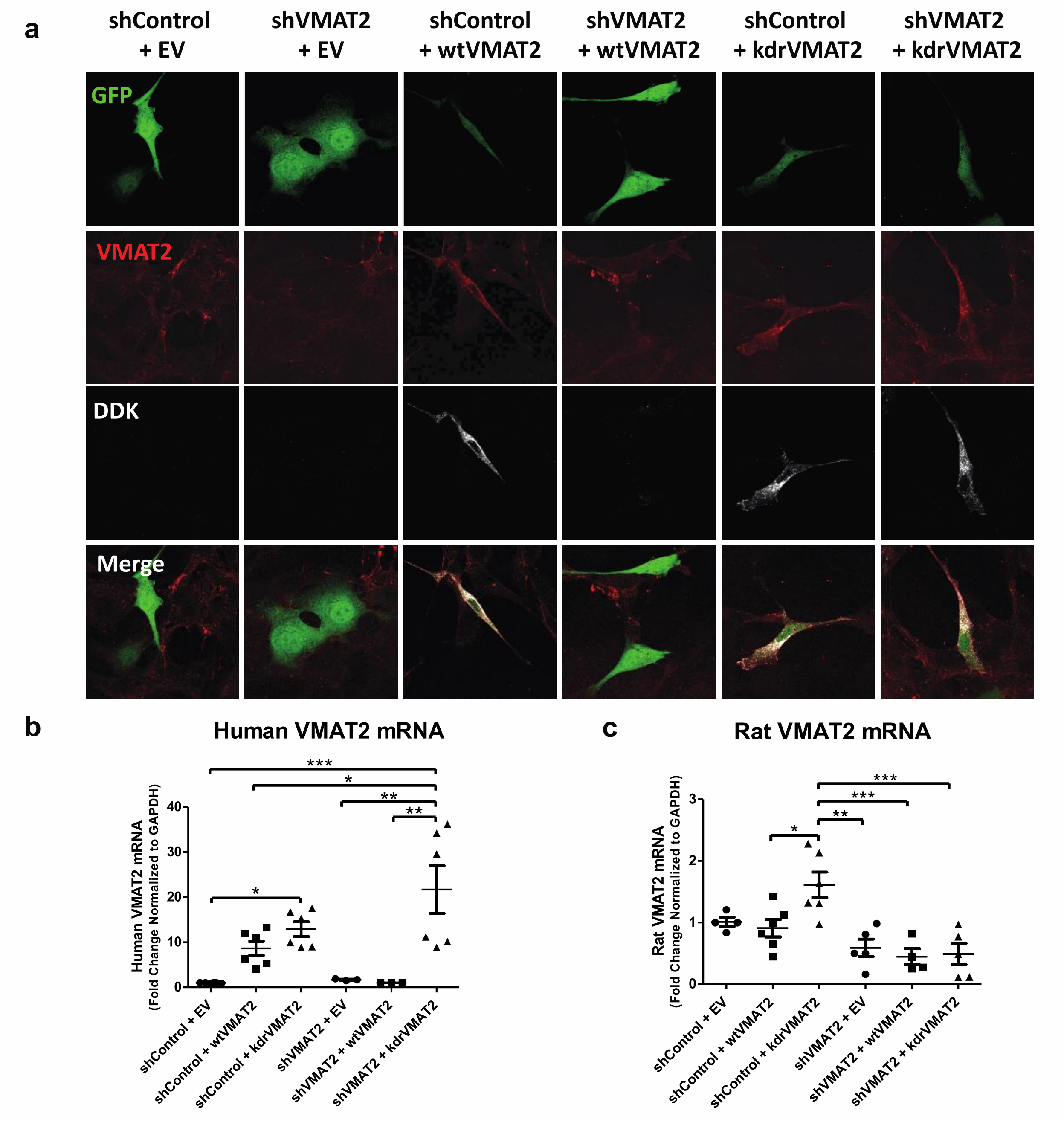


Supplementary Figure 7. Reintroduction of exogenous VMAT2 with a knock-down resistant construct. A. Immunocytochemistry performed in RCSN-3 cells transfected with empty vector (EV), shControl, shVMAT2, wild type VMAT2 (wtVMAT2), or knock-down resistant VMAT2 (kdrVMAT2) with immunolabeling for GFP (green), VMAT2 (red), and DDK (white). B. Human VMAT2 mRNA normalized to GAPDH analyzed by qtPCR (one-way ANOVA with Tukey's post-hoc test, n=4-6 experimental replicates, *p<0.05, **p<0.01, ***p<0.001). C. Rat VMAT2 mRNA normalized to GAPDH analyzed by qtPCR (one-way ANOVA with Tukey's post-hoc test, n=3-6 experimental replicates, *p<0.05, **p<0.01, ***p<0.001).

**Supplementary Table 1. Primers used for qt-PCR**

| Gene | Organism | Sequence (Forward 5'-3') | Item # |
|---|---|---|---|
| SLC18A2 | Rat | CATTTTATGCATCCCCTTTG | VRPS-5656 |
| SLC18A2 | Human | ATGAGTTTGTGGGGAAGACG | VHPS-8457 |
| GAPDH | Rat | AGACAGCCGCATCTTCTTGT | VRPS-6986 |
| GAPDH | Human | GAGTCAACGGATTTGGTCGT | VHPS-3541 |

**Supplementary Table 2. Summary of cohorts used in each analysis**

| Figure | Analysis | Viral treatment | Viral titer (genome copies/mL) | Number of animals | Male/Female | Time post-viral transduction |
|---|---|---|---|---|---|---|
| Figure 1. | Nigral VMAT2 and TH | AAV2-shVMAT2 AAV2-shControl | $3.56x10^{12}$ | 5 | Male | 6 weeks |
| | | | $3.56x10^{12}$ | 5 | | 6 weeks |
| Figure 2. | Striatal VMAT2 and TH | AAV2-shVMAT2 | $3.56x10^{12}$ | 5 | Male | 6 weeks |
| | Neurochemistry | AAV2-shVMAT2 | $3.56x10^{12}$ | 4 | Male | 8 weeks |
| Figure 3. | Stereology | AAV2-shVMAT2 | $3.56x10^{12}$ | 5 | Male | 6 weeks |
| | Stereology | AAV2-shVMAT2 | $3.56x10^{12}$ | 5 | Female | 6 weeks |
| | Stereology | AAV2-shControl | $3.56x10^{12}$ | 5 | Male | 6 weeks |
| | Behavior | Ipsilateral AAV2-shVMAT2 | $3.56x10^{12}$ | 4 | Male | 20 weeks |
| | | Contralateral AAV2-shControl | $1.53x10^{13}$ | | | |
| Figure 4. | LRRK2 PLA | AAV2-shVMAT2 | $3.56x10^{12}$ | 6 | Male | 6 weeks |
| | Phosphorylated Rab10 | AAV2-shVMAT2 | $3.56x10^{12}$ | 4 | Male | 6 weeks |
| Figure 5. | α-synuclein | AAV2-shVMAT2 | $3.56x10^{12}$ | 3 | Male | 6 weeks |
| Figure 6. | Stereology and nigral VMAT2 | AAV2-shVMAT2 + AAV2-GFP | $2.0x10^{12}$ and $2.0x10^{12}$ | 7 | Male | 6 weeks |
| | | AAV2-shVMAT2 + AAV2-kdrVMAT2 | $2.0x10^{12}$ and $2.0x10^{12}$ | 5 | | 6 weeks |
| Supplementary Figure 2. | Nigral VMAT2 | AAV2-shVMAT2 | $3.56x10^{12}$ | 5 | Female | 6 weeks |
| Supplementary Figure 3. | Neurochemistry | AAV2-shVMAT2 | $3.56x10^{12}$ | 3 | Male | 4 weeks |
| | | | | 3 | Male | 12 weeks |
| Supplementary Figure 3. | Stereology | AAV2-shVMAT2 | $3.56x10^{12}$ | 3 | Male | 4 weeks |
| | | | | 3 | Male | 8 weeks |
| | | | | 3 | Male | 12 weeks |
| Supplementary Figure 4. | 4HNE and 3NT | AAV2-shVMAT2 AAV2-shControl | $1.78x10^{12}$ | 9 | Male | 6 weeks |
| | | | $3.56x10^{12}$ | 3-5 | | 6 weeks |
| Supplementary Figure 5. | LRRK2 PLA | AAV2-shVMAT2 | $3.56x10^{12}$ | 6 | Female | 6 weeks |
| | Phosphorylated Rab10 | AAV2-shVMAT2 | $3.56x10^{12}$ | 6 | Female | 6 weeks |
| | Phosphorylated Rab10 | AAV2-shControl | $3.56x10^{12}$ | 4 | Male | 6 weeks |
| Supplementary Figure 6. | α-synuclein | AAV2-shControl | $3.56x10^{12}$ | 4 | Male | 6 weeks |
