# Supplementary material for: Acquired dysregulation of dopamine homeostasis reproduces features of Parkinson’s disease
Source: NPJ Parkinsons Dis. 2020 Nov 13;6:34. doi: 10.1038/s41531-020-00134-x (PMC7666186; doi:10.1038/s41531-020-00134-x)
Supplement: Supplementary file 2 — Reporting Summary FLAT [file 41531_2020_134_MOESM2_ESM.pdf]

nature research

Corresponding author(s): Teresa G Hastings
Meghan L Bucher

Last updated by author(s): August 19, 2020

Reporting Summary

Nature Research wishes to improve the reproducibility of the work that we publish. This form provides structure for consistency and transparency in reporting. For further information on Nature Research policies, see Authors & Referees and the Editorial Policy Checklist.

Statistics

For all statistical analyses, confirm that the following items are present in the figure legend, table legend, main text, or Methods section.

n/a	Confirmed	
☐	☒	The exact sample size (*n*) for each experimental group/condition, given as a discrete number and unit of measurement
☐	☒	A statement on whether measurements were taken from distinct samples or whether the same sample was measured repeatedly
☐	☒	The statistical test(s) used AND whether they are one- or two-sided<br>*Only common tests should be described solely by name; describe more complex techniques in the Methods section.*
☒	☐	A description of all covariates tested
☐	☒	A description of any assumptions or corrections, such as tests of normality and adjustment for multiple comparisons
☐	☒	A full description of the statistical parameters including central tendency (e.g. means) or other basic estimates (e.g. regression coefficient) AND variation (e.g. standard deviation) or associated estimates of uncertainty (e.g. confidence intervals)
☐	☒	For null hypothesis testing, the test statistic (e.g. *F*, *t*, *r*) with confidence intervals, effect sizes, degrees of freedom and *P* value noted<br>*Give P values as exact values whenever suitable.*
☒	☐	For Bayesian analysis, information on the choice of priors and Markov chain Monte Carlo settings
☒	☐	For hierarchical and complex designs, identification of the appropriate level for tests and full reporting of outcomes
☒	☐	Estimates of effect sizes (e.g. Cohen's *d*, Pearson's *r*), indicating how they were calculated

Our web collection on statistics for biologists contains articles on many of the points above.

Software and code

Policy information about availability of computer code

Data collection	Software used for data collection: Fluoview 1000 software (Melville, NY)
Data analysis	Software used to analyze data include Fluoview 1000 software (Melville, NY), GraphPad Prism software (V. 5.01), and Nikon NIS-Elements Advanced Research software (Version 4.5, Nikon, Melville, NY).

For manuscripts utilizing custom algorithms or software that are central to the research but not yet described in published literature, software must be made available to editors/reviewers. We strongly encourage code deposition in a community repository (e.g. GitHub). See the Nature Research guidelines for submitting code & software for further information.

Data

Policy information about availability of data

All manuscripts must include a data availability statement. This statement should provide the following information, where applicable:

- Accession codes, unique identifiers, or web links for publicly available datasets
- A list of figures that have associated raw data
- A description of any restrictions on data availability

The data generated during and/or analyzed during the current study are available within the paper and supplementary files.

Field-specific reporting

Please select the one below that is the best fit for your research. If you are not sure, read the appropriate sections before making your selection.

☒ Life sciences ☐ Behavioural & social sciences ☐ Ecological, evolutionary & environmental sciences

Life sciences study design

All studies must disclose on these points even when the disclosure is negative.

Sample size	No sample-size calculation was performed. Sample sizes were determined on the number of animals used in previous studies performed by our group (Zharikov et al. 2015, Zharikov et al. 2018, De Miranda et al. 2018)
Data exclusions	No data were excluded.
Replication	Findings were replicated in independent cohorts receiving either the same, or similar viral treatment with variations in viral titer or post-transduction time period.
Randomization	Animals were randomly assigned to experimental groups.
Blinding	As the nature of the experiments required identifying virally-transduced dopaminergic neurons (determined by coexpression of TH and GFP staining), intensity and number of objects analyses were obtained by circling ROIs around virally-transduced neurons (as identified as being both TH- and GFP-positive) while blinded to the protein of interest. In non-transduced tissue, ROIs were drawn around TH-positive neurons while blinded to the protein of interest.

Reporting for specific materials, systems and methods

We require information from authors about some types of materials, experimental systems and methods used in many studies. Here, indicate whether each material, system or method listed is relevant to your study. If you are not sure if a list item applies to your research, read the appropriate section before selecting a response.

Materials & experimental systems

n/a	Involved in the study
☐	☒ Antibodies
☐	☒ Eukaryotic cell lines
☒	☐ Palaeontology
☐	☒ Animals and other organisms
☒	☐ Human research participants
☒	☐ Clinical data

Methods

n/a	Involved in the study
☒	☐ ChIP-seq
☒	☐ Flow cytometry
☒	☐ MRI-based neuroimaging

Antibodies

Antibodies used

GFP Millipore MAB3850
TH Millipore AB152
VMAT2 Santa Cruz sc-7721
4HNE Abcam AB46545
3NT Santa Cruz sc-32757
α-synuclein BD Biosciences 610787
Phosphorylated α-synuclein Abcam AB1253
Phosphorylated Rab10 Abcam ab230261
MAP2 Millipore MAB378
TOM20 Santa Cruz sc-11415
LRRK2 UC Davis N241A/34
Phosphorylated LRRK2 Abcam AB203181

Validation

GFP: Well established antibody with validation on Millipore website and extensive citations including Zharikov et al. 2015

TH: Well established antibody with validation on Millipore website and extensive citations including Zharikov et al. 2015
VMAT2: Supplemental Figure 1 demonstrating decreased immunoreactivity following shRNA against VMAT2

4HNE: Via Abcam.com "Validated in WB, ELISA, IHC and tested in Species independent. Cited in 142 publication(s). Independently reviewed in 18 review(s)."
3NT: Extensive citation history (86 citations) including De Miranda et al. 2018

α-synuclein: Extensive citation history including De Miranda et al. 2019
Phosphorylated α-synuclein: Extensive citation history including De Miranda et al. 2019

Phosphorylated Rab10 Abcam ab230261: Knock-out validated via Abcam.com
MAP2: Well established antibody with validation on Millipore website and extensive citations including Tapias et al. 2014

TOM20: Previously characterized in Di Maio et al. 2016

	LRRK2: Previously characterized in Di Maio et al. 2018<br><br>Phosphorylated LRRK2: Previously characterized in Di Maio et al. 2018

Eukaryotic cell lines

Policy information about cell lines

Cell line source(s)	RCSN-3 cells were obtained through a material transfer agreement between the University of South Florida University of Chile.
Authentication	The cell line was not authenticated upon arrival.
Mycoplasma contamination	The cells were not tested for mycoplasma contamination.
Commonly misidentified lines<br>(See ICLAC register)	No commonly misidentified cell lines were used in this study.

Animals and other organisms

Policy information about studies involving animals; ARRIVE guidelines recommended for reporting animal research

Laboratory animals	Male and female adult Lewis rats were used in this study.
Wild animals	The study did not involve wild animals.
Field-collected samples	This study did not involve samples collected from the field.
Ethics oversight	Experimental approval was obtained through the Institutional Animal Care and Use Committee at the University of Pittsburgh.

Note that full information on the approval of the study protocol must also be provided in the manuscript.
